# Male pheromone polymorphism and reproductive isolation in populations of *Drosophila simulans*

**DOI:** 10.1002/ece3.342

**Published:** 2012-09-08

**Authors:** Gwénaëlle Bontonou, Béatrice Denis, Claude Wicker-Thomas

**Affiliations:** CNRS UPR 9034, Université de Paris Sud91198, Gif sur Yvette, France

**Keywords:** Desiccation resistance, hydrocarbons, reproductive isolation, temperature

## Abstract

The dominant cuticular hydrocarbons (HC) in *Drosophila simulans* are 7-tricosene (7-T) and 7-pentacosene (7-P). The 7-T is the major HC in East Africa and in other continents. In West Africa, *D. simulans* is very rare and displays 7-P as the major compound. We studied three *D. simulans* strains from Egypt (Eg), Sao-Tome (ST), and Cameroon (Cam), with 7-T, intermediary or 7-P phenotypes. HC profiles of ST and Cam female differed slightly from corresponding male profiles; females had more 7-T and less 7-P. Varying temperature affected all HCs (even those with 27 and 29 carbons)-not just 7-T and 7-P; there was no clear relationship between HC phenotype and resistance to desiccation. We report reproductive isolation between Eg and ST and Eg and Cam (but not between ST and Cam), which is due to Eg and Cam female preferences for their own males. In conclusion, our findings do support divergence of *D. simulans* populations from West Africa for both pheromonal profile and mating preference.

## Introduction

All *Drosophila* species have abundant long-chain cuticular hydrocarbons (HCs) that act both to prevent desiccation and as sex pheromones during courtship (Dillwith et al. 1981; Jallon 1984). In the *D. melanogaster* subgroup, specific female HCs are involved in reproductive isolation and speciation, such as between *D. melanogaster* and *D. simulans* (Coyne 1996a), *D. sechellia* and *D. simulans* (Coyne 1996b), *D. mauritiana* and *D. sechellia* (Coyne and Charlesworth 1997), and even *D. melanogaster* populations with different pheromone phenotypes (Wu et al. 1995). Reproductive isolation and speciation can also be due to differences in male HCs, as described between *D. yakuba* and *D. santomea* (Mas and Jallon 2005) and pheromonal *D. melanogaster* races (Grillet et al. 2012). In other subgroups, such as the *D. montium* group, there is evidence that male HCs play a role in speciation between *D. serrata* and *D. birchii* (Blows and Allan 1998; Howard et al. 2003).

The *D. melanogaster* subgroup consists of nine species, all originating from Africa. About half of the total hydrocarbons are 7-tricosene (C23:1; 7-T) or 7-pentacosene (C25:1; 7-P), and the chemical composition is similar in males of all species except *D. erecta* (Jallon and David 1987). In contrast, the chemical composition of female HCs is species-dependent; both male and female *D. yakuba*, *D. santomea*, *D. teissieri*, *D. orena*, *D. mauritiana*, and *D. simulans* have the same HC pattern, although there is sexual dimorphism in the relative HC abundance (Ferveur 1991; Ferveur and Jallon 1993; Sharma et al. 2012a). Females of two species, *D. melanogaster* and *D. sechellia*, have dienes in the 27 and 29 carbons that act as sex pheromones. *D. erecta* is characterized by a peculiar long-chain (C31–C33) cuticular HC pattern in both males and females (Jallon and David 1987).

The HC composition is relatively homogenous within all *Drosophila* species, except in *D. melanogaster* and *D. simulans* cosmopolitan species (Jallon and David 1987). There is a geographic HC polymorphism in *D. melanogaster* females that affects the composition of the dienes (Ferveur et al. 1996). Studies have suggested that this polymorphism led to premating isolation between Zimbabwe flies and the other populations (Begun and Aquadro 1993; Wu et al. 1995; Hollocher et al. 1997a,b). Another geographic HC polymorphism has been described in males: while 7-T is usually the dominant male HC, males in some African strains are richer in 7-P (Jallon 1984). A large-scale study involving 85 *D. melanogaster* populations found a significant correlation between the male 7-T/7-P ratio and latitude, mean temperature, and vapor pressure (Rouault et al. 2001, 2004). This male pheromone polymorphism is also responsible for reproductive isolation between pheromonal races (Grillet et al. 2012; Bontonou et al., unpublished data).

The 7-T is usually the major HC in *D. simulans*. However, rare populations have been found in West Africa, with higher levels of 7-P (Rouault et al. 2001). The 7-T is the main female pheromone; it induces wing vibration in *D. simulans* males from the Seychelles (Jallon 1984). However, the roles of 7-P in all strains and of 7-T in 7-P strains remain unknown. *Drosophila simulans* females – rich in 7-P- were reported to stimulate courtship behavior in both males rich in 7-P and those rich in 7-T rich. Cobb and Jallon (1990) suggested that 7-P might also play a role in stimulating courtship in *D. simulans* males, perhaps through synergistic effects.

**Figure 1:**
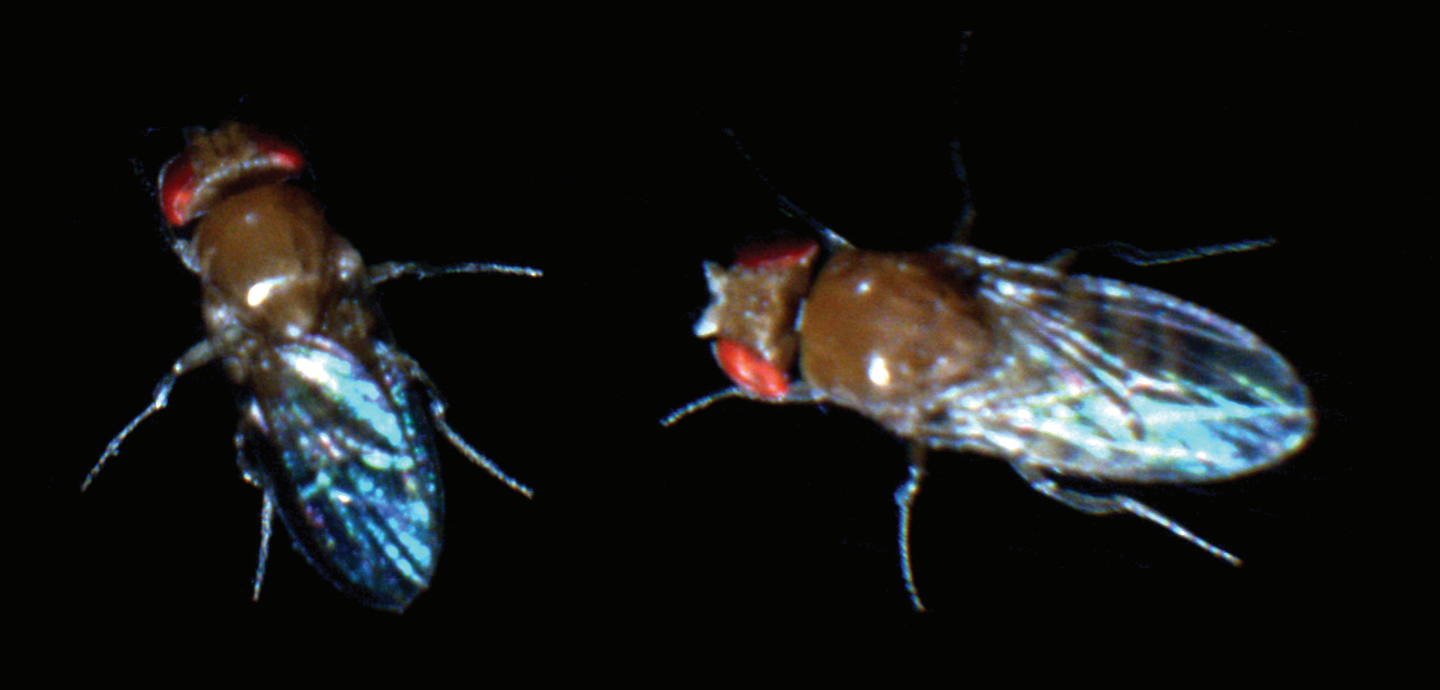
Courtship behavior in *Drosophila simulans* (Cam strain).

We wondered whether the difference in pheromone composition has led to reproductive isolation between *D. simulans* populations rich in 7-T and those rich in 7-P, and the role of temperature in determining the 7-T/7-P ratio. We used three African *D. simulans* strains, each with different HC profiles (Fig. 1). We first show that differences in HC ratios can lead to reproductive isolation that is caused by females discriminating between the male pheromones. We then investigated the effect of temperature on HC profiles and showed that 7-P was higher and 7-T lower at 25°C than at 21°C in 7-P (but not 7-T) strains. We also found that the absolute amount of HCs is more important than the relative HC composition in resistance to desiccation. This study is the first to suggest a role of pheromones in reproductive isolation between *D. simulans* populations.

## Materials and Methods

### Strains

We analyzed the HC profiles of 37 strains originating from 16 different locations (Table S1). The three following strains were used in this study: Al12 from Egypt (named Eg in the study), high in 7-T, BS3 from Sao Tome (named ST), intermediate 7-T/7-P ratio, and 386-11 from Cameroon (named Cam), high in 7-P. Flies were reared on standard yeast/cornmeal/agar medium at 25°C with a 12/12-h light/dark cycle.

### Hydrocarbon analyses

At emergence, flies were lightly anesthetized with CO_2_ and kept in groups of 10 on standard medium at 21, 25, or 29°C. Development is temperature-dependent and HC maturation takes about 6 days at 21°C and about 4 days at 25 and 29°C. As a consequence, HC was extracted on day 5 from usually 10 individuals at 25 or 29°C and on day 7 from individuals at 21°C. Samples were prepared for gas chromatography by placing one fly in a microvial insert with 100 μL of heptane and 500 ng n-C26 (as an internal standard) for 5 min then removing the fly and capping with a PTFE (polytetrafluoroethylene) cap. We stored the samples at 4°C until they were analyzed on the gas chromatograph. We used a split injector (split ratio 40/1) to inject 5 μL of each sample into a Perichrom Pr200 gas chromatograph, fitted with a flame-ionization detector, with a BP-1 capillary column (SGE, 25 m long, 220 μm in diameter and 0.1 μm ID) and hydrogen as the carrier gas (25 cm/s velocity). The injector and detector temperatures were 250 and 26°C, respectively. The oven-temperature started at 180°C, ramped at 3°C/min to 300°C, for a total run of 40 min. The data were automatically computed and recorded using Winilab.

### Cuticular hydrocarbons and multivariate analyses

Each peak with a chain length between 23 and 29 carbons was quantified using Winilab. We analyzed 14 HCs in male flies, all with a chain length between 23 and 29 carbons. Peak areas were calculated as a proportion of total HC content. We calculated total HC quantities by summing the area under each peak and normalized this value by dividing by the abundance of the internal C26 standard. We calculated the relative abundance of each HC for each individual by dividing the area under each peak by the total area under all peaks. This corrected for nonbiological sources of HC variation among samples (Blows and Allan 1998). Data are presented as means ± SEM (*n* = 10 for all tests).

To study the impact of temperature (21, 25, or 29°C) on HC synthesis, proportions were arcsine-square root-transformed and one-way analyses of variance (ANOVAs) were performed separately for each HC. Log-contrasts were calculated to compare HC profiles of males from different strains at 25°C. This transformation removes the unit-sum constraint associated with proportional data (Blows and Allan 1998) and reduces the number of traits by one. We used the proportion of 2-Me-C22 as the denominator in males and (Z)-5-C23:1 in females: log-contrast(HC_n_) = log_10_(proportion[HC_n_]/proportion[2-Me-C22 or (Z)-5-C23:1]), resulting in 13 log-contrasts variables. Each log-contrast HC was analyzed separately by ANOVAs. Significant differences among groups detected by ANOVA were analyzed using Tukey's post-test (including a correction for multiple comparisons) as the post-hoc test to identify groups exhibiting statistically significant differences. All statistical analyses were performed using R version 2.13.1 (free software available at http://www.r-project.org/. CRAN mirror used: CICT, Toulouse, France http://cran.cict.fr/).

### Desiccation resistance

One hundred male or female flies from each experimental strain were briefly anesthetized with CO_2_ then transferred to empty plastic tubes sealed with a cotton plug permitted the entry of dry air. The tubes were placed in a hermetically sealed box containing silica gel to maintain low humidity, and the entire box placed in an incubator at 25°C. Mortality was recorded every 30 min. We defined the median survival interval (MSD) as the intercept of the survival curve and the horizontal 0.5 level (Rouault et al. 2004). We expressed MSDs as decimal hour and presented them with 95% confidence intervals.

### Mating experiments

At emergence, flies were lightly anesthetized with CO_2_, separated by sex, and kept in groups of 10 flies on standard medium for 6 days at 25°C. We ran the courtship assays at approximately the same time each morning. The glass observation chamber used for single-pair tests was a watch glass of 28 mm diameter and 5 mm internal height, placed on a glass plate.

### No-choice experiment tests

We introduced a female into the observation chamber and left her for 1 min before introducing the male. Ninety-nine percent of mating pairs copulated within 60 min. We recorded courtship latency and copulation latency (time between when the male was introduced into the female-containing observation chamber and when courtship or copulation occurred); Males and females from the three strains were paired in the nine possible combinations (*n* = 30 for all tests). We used Kruskal–Wallis tests followed by multiple pairwise comparison (pgirmess package on R, kruskalmc function) to compare courtship and copulation latencies between mating types.

### Male-, female-, and multiple-choice tests

We carried out all assays for male-, female-, and multiple-choice tests until 50 copulations had occurred, but never for more than one hour. For statistical purposes, we also recorded the number of flies that did not mate. We cut a small portion of one wing (alternatively right or left) of all the flies to allow us to differentiate flies of different strains in mate-choice tests.

In male-choice tests, we transferred a single male onto a glass observation chamber with two females from different strains. The trial ended once one female had copulated with the male. In female-choice tests, we transferred a single female onto a glass observation chamber with two males from different strains. The trial ended once the female had copulated with one of the males.

Multiple-choice tests have been also performed because they are more sensitive at detecting assortative mating than no-choice tests and they permit both male and female choice to contribute to assortative mating (Kwan and Rundle 2010). Trials were performed in rearing tubes with food medium. For each replicate mating trial, two males and two females (one male and one female of each strain) were introduced into a fresh food vial. The trial ended when the first copulation occurred. For each mate-choice design combination, we used JMating Software to calculate the pair total index (PTI) and the index of sexual isolation (*I*_PSI_; Rolan-Alvarez and Caballero 2000). PTI is the ratio of observed mating frequencies to those expected if mating between strains were random. It combines the contribution of sexual selection and sexual isolation for each pair type. PTI varies between zero and infinity, with a value of one indicating that species show no sexual isolation or do not differ in mating propensity. The PTI is the best estimate of true mating preferences when there are no detectable differences in mating propensity between tested species. *I*_PSI_ is an index, that describes overall sexual isolation in experiments. *I*_PSI_ varies from −1 to 1, where −1 represents complete disassortative mating, 0 represents random mating, and 1 represents complete assortative mating (complete sexual isolation). We determined statistical significance of *I*_PSI_ and PTI by bootstrapping 10,000 times in JMating.

## Results

### Comparison of the hydrocarbons between strains

The percentages of 7-T and 7-P of the 37 strains originating from 16 different locations are given in Table S1. We found similar HC compositions of the different strains after their maintenance in the laboratory for several years. Three groups, concerning the HC profiles, were obtained: the first group, including the six non-African populations and 15 African populations, high in 7-T; the second group, composed of the two strains from Sao-Tome, with intermediate 7-T/7-P ratio; the third group, composed of the four Cameroon populations, high in 7-P. In this study we chose a continental strain, AL12 (Eg) for the first group, which has a high 7-T/7-P ratio and was collected quite recently. The strains belonging to the second and third group were homogenous and two of them were arbitrarily used for the experiments.

**Figure 2:**
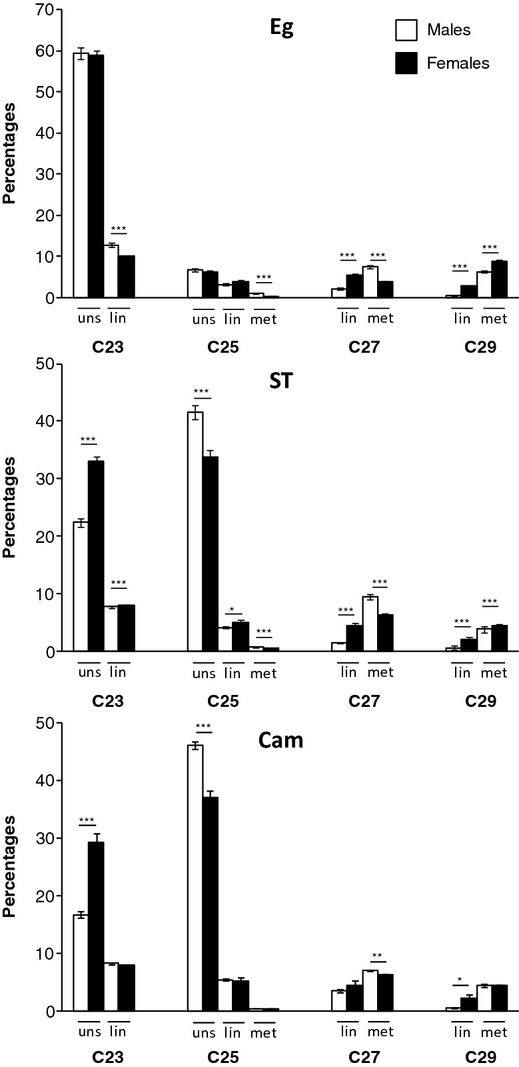
Hydrocarbons in males and females of the three strains. Bars represent means ± SEM (*n* = 10). Means with *, **, *** were significantly different at *P* = 0.05, 0.01, and 0.001, respectively; uns, unsaturated hydrocarbons; met, methylated hydrocarbons; lin, saturated linear hydrocarbons.

Total amounts of HCs were 22 and 28% lower in ST and Cam males, than in Eg males (Table 1). The ST and Cam females had similar HC amounts, Eg females has 42% fewer HCs than Cam (Table 2). From the HC profiles, we could identify 20 HC peaks using gas chromatography. All of them were shared by males and females from all three strains. We removed six HCs before analysis (corresponding to (Z)-9-C27:1, (Z)-7-C27:1, (Z)-7-C27:1, (Z)-9-C29:1, (Z)-7-C29:1, (Z)-7-C29:1) because of their low and variables quantities. In each population, males and females had similar HC phenotypes even when the relative HC abundance differed (Fig. 2). In all strains females had more 7-T and more total HCs than males. The three strains had different HC profiles (Tables 1 and 2). Eg flies had two times more 7-T and ten times less 7-P than ST and Cam flies. The HC profile of ST flies was intermediate between those of Eg and Cam. The ratio of 7-T/7-P in ST males (0.59) differed significantly from that of Eg males (12.7) and Cam males (0.37), although 7-T and 7-P values did not differ significantly between ST and Cam males, probably because of the denominator choice. Females of the three strains differed significantly in the proportion of 7-P.

**Table 1:** Analysis of differences between the HC profiles of males from strains at 25°C

HC	*F*	*P*_Eg-Cam_	*P*_ST-Cam_	*P*_ST-Eg_	Eg	ST	Cam
HC (ng/fly)	6.19	**<.01**	0.79	**0.03**	1577±78	1217±115	1128±91
(Z)-9-C23:1	15.33	**<.001**	0.98	**<.001**	5.24±0.21	1.12±0.10	1.05±0.08
(Z)-7-C23:1	6.62	**<.01**	0.59	**0.04**	52.01±1.13	20.42±0.56	14.93±0.47
(Z)-5-C23:1	4.95	**0.03**	0.96	**0.04**	2.08±0.08	0.88±0.10	0.76±0.05
C23	0.82	0.57	0.99	0.48	12.78±0.42	7.77±0.21	8.26±0.24
2-Me-C24	1.8	0.25	0.26	1.00	0.94±0.19	0.77±0.09	0.44±0.07
(Z)-9-C25:1	4.84	**0.03**	0.97	**0.04**	2.62±0.13	5.33±0.18	5.88±0.16
(Z)-7-C25:1	30.78	**<.0001**	0.94	**<.0001**	4.08±0.17	34.55±0.87	39.15±0.44
(Z)-5-C25:1	35.38	**<.0001**	0.51	**<.0001**	0.05±0.02	1.66±0.09	1.09±0.04
C25	2.19	0.12	0.71	0.43	3.09±0.27	4.14±0.18	5.45±0.16
2-Me-C26	0.76	0.98	0.61	0.5	7.40±0.44	9.55±0.44	7.02±0.15
C27	2.95	0.14	0.1	0.99	2.10±0.32	1.51±0.13	3.46±0.26
2-Me-C28	0.81	0.77	0.85	0.43	6.20±0.26	3.88±0.53	4.47±0.29
C29	0.48	0.72	0.65	1.00	0.45±0.10	0.66±0.36	0.59±0.09

HC identities are given in the first column; elemental composition is listed as the carbon chain length followed by the number of double bonds. HCs are expressed in ng/fly (first line) and in percentages. Statistical analyses were performed using a one-way ANOVA followed by Tukey's multiple comparison post-hoc test. *P*-values in bold indicate significant HC variations with strains after multiple test correction. The last three columns give the mean±SEM (*n* = 10) of HCs produced by 5-day-old males at 25°C.

**Table 2:** Analysis of differences between the HC profiles of females from strains at 25°C

HC	*F*	*P*_Eg-Cam_	*P*_ST-Cam_	*P*_ST-Eg_	Eg	ST	Cam
HC (ng/fly)	6.00	**<.01**	0.55	0.07	2141±167	2767±210	3044±187
(Z)-9-C23:1	25.28	**<.0001**	0.48	**<.0001**	2.1±0.08	0.72±0.1	0.76±0.11
(Z)-7-C23:1	115.96	**<.0001**	0.77	**<.0001**	54.83±1.1	30.25±0.63	26.67±1.42
(Z)-5-C23:1					2.12±0.08	1.96±0.06	1.83±0.15
C23	3.94	0.45	0.28	**0.03**	9.97±0.22	8.04±0.16	7.97±0.16
2-Me-C24	4.33	0.16	0.61	**0.02**	0.28±0.05	0.63±0.12	0.45±0.07
(Z)-9-C25:1	11.72	**<.001**	0.13	**0.03**	2.33±0.08	3.56±0.15	3.87±0.21
(Z)-7-C25:1	283.42	**<.0001**	**0.04**	**<.0001**	3.87±0.28	29.81±0.98	32.6±1.09
(Z)-5-C25:1	5.43	**0.01**	0.59	0.09	0.03±0.01	0.41±0.04	0.59±0.04
C25	3.22	**0.04**	0.43	0.43	3.84±0.28	5.07±0.38	5.28±0.54
2-Me-C26	15.59	**<.0001**	0.35	**0.001**	3.80±0.24	6.36±0.32	6.24±0.17
C27	1.08	0.72	0.78	0.33	5.38±0.4	4.46±0.45	4.49±0.74
2-Me-C28	17.88	**<.001**	0.65	**<.0001**	8.89±0.4	4.59±0.26	4.36±0.24
C29	1.36	0.45	0.94	0.28	2.68±0.29	2.05±0.44	2.2±0.7

HC identities are given in the first column; elemental composition is listed as the carbon chain length followed by the number of double bonds. HCs are expressed in ng/ fly (first line) and in percentages. Statistical analyses were performed using a one-way ANOVA followed by Tukey's multiple comparison post-hoc test. *P*-values in bold indicate significant HC variations with strains after multiple test correction. The last three columns give the mean±SEM (*n* = 10) of HCs produced by 5-day-old females at 25°C.

### Resistance to desiccation

**Figure 3:**
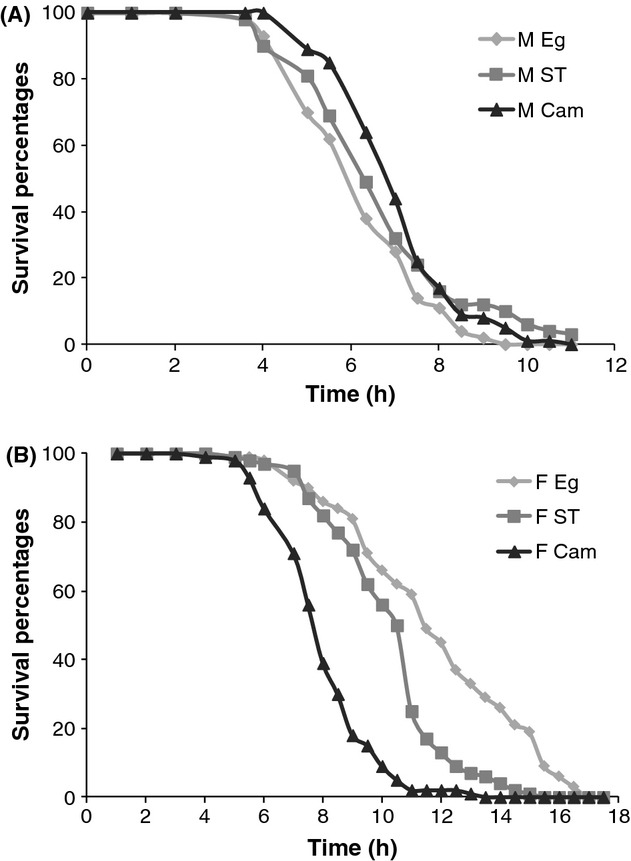
Survival percentages of male and female flies from different strains, as a function of exposure time to desiccation stress at 25°C (*n* = 100).

Females were 1.5 times more resistant to desiccation than males (Fig. 3); their median survival duration (MSD) was 9.9 h (6.4 h for males; *n* = 100). Males from all strains showed similar resistance to desiccation; Cam females were much less resistant to desiccation (MSD of 7.7 h) than ST and Eg females (10.5 and 11.5 h).

### Influence of temperature on HC profiles

**Figure 4:**
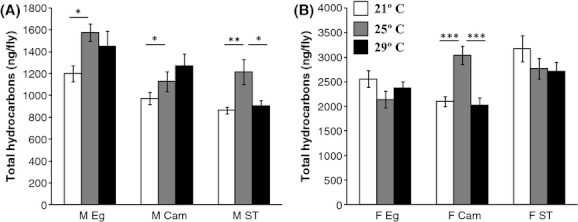
No-choice mating tests: courtship and copulation latencies in fly pairs from the same or from different strains of *Drosophila simulans*. Each fly pair is represented with the female listed first. Each bar represents mean ± SEM of 30 trials. Different letters above bars indicate significant differences between means based on Kruskal–Wallis tests (*P* = 0.05) followed by a multiple comparison test.

Analyses of differences between the HC profiles at 21, 25, and 29°C are shown in Tables S2–S4 for Eg, ST, and CAM males and in Tables S5–S7 for Eg, ST, and CAM females, respectively. In all males and in Cam females, the total HC amounts were significantly lower at 21 than at 25°C (Fig. 4) and lower at 29 than at 25°C in ST males and Cam females. The effects on each hydrocarbon differed with strain and sex. In all flies, there was a positive correlation between 2-Me-C28 and a negative correlation between 2-Me-C26 and increased temperature. A shift of temperature from 21 to 25°C was followed by an increase in 7-P and decrease in 7-T in all males and Eg females. Also, except for Cam females, there were fewer C23 monoenes and more C25 monoenes at 25 than at 21°C.

### Mating experiments

**Figure 5:**
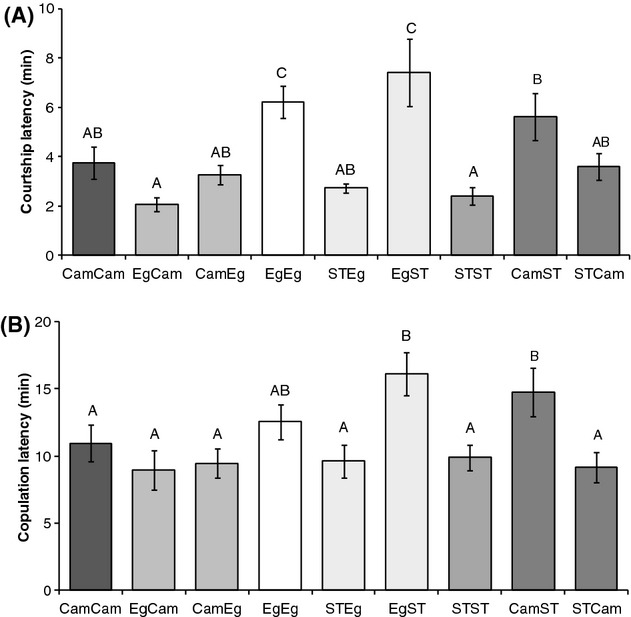
Effect of a temperature (7 days at 21°C or 5 days at 25 or 29°C) on the absolute quantities of hydrocarbons in Eg, ST and Cam males (A) and females (B). Each bar represents mean ± SEM (*n* = 10). *, **, and *** above bars indicate significant differences (one-way ANOVA, followed by Tukey's multiple comparison post-hoc test, *P* = 0.05, 0.01 and 0.001, respectively) between means.

We used a classical no-choice test to study courtship and copulation latencies of flies from the three populations for all among the nine possible cross combinations (Fig. 5). Intra-strain courtship latency varied significantly among the three laboratory strains (Kruskal–Wallis χ² = 20.13, *P* < 0.001), unlike the copulation latency (Kruskal–Wallis χ² = 2.15, *P* = 0.34). Eg flies had the longest intra-strain courtship (6.2 min vs. 3.7 and 2.4 min for Cam and ST, respectively). Courtship between Eg males with Eg females took longer than with ST or Cam females but copulation took about the same amount of time for all flies. ST males paired with females from other strains had longer courtship and copulation and, for Cam males, there was no significant variation in courtship and copulation for any pairing with females of any strain.

**Figure 6:**
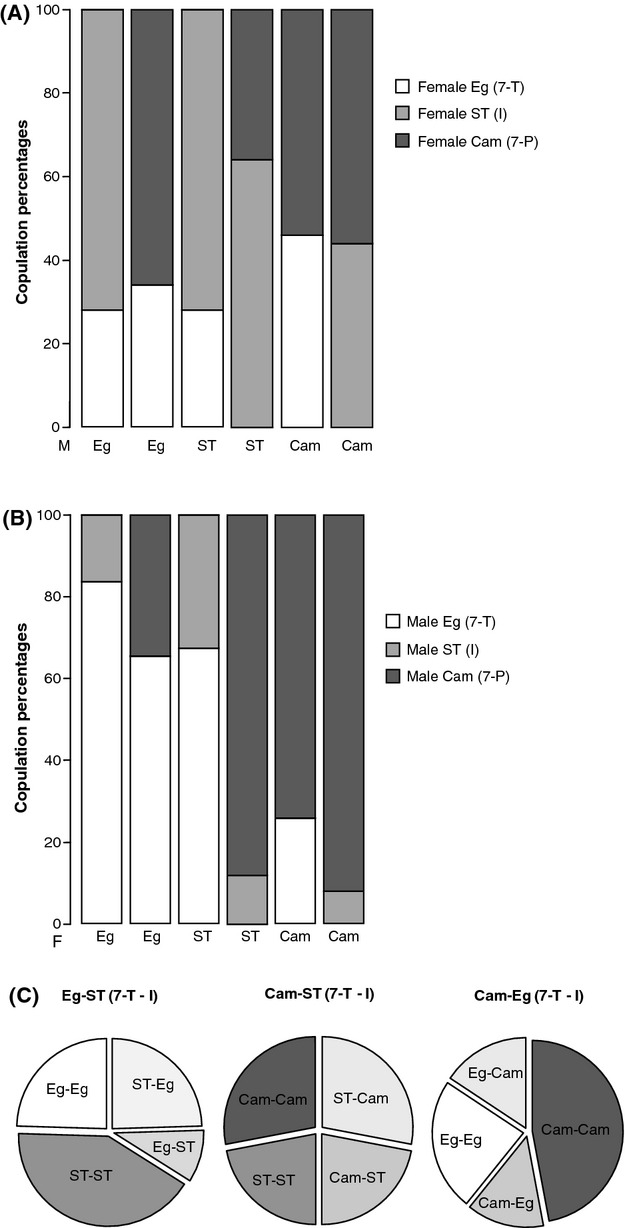
(A) Male-choice mating tests. A single male was transferred onto a glass observation chamber with one conspecific female and one heterospecific female. Each bar corresponds to the percentage of copulations between males and available females. The male line is noted on the *X*-axis, *n* ≥ 50. (B) Female-choice mating tests. A single female was transferred onto a glass observation chamber with one conspecific male and one heterospecific male. Each bar corresponds to the percentage of copulations between females and available males. The female line is noted on the *X*-axis, *n* ≥ 50. (C) Multiple-choice experiments. Pie chart diagrams represent the proportion of copulations that occurred between flies from two different lines. Types of pairings are indicated above each diagram. Copulation mating pairs are noted inside each section, female given first, *n* ≥ 50.

The distribution of effective matings in female-, male-, and multiple-choice tests is presented in Fig. 6A–C. The numbers of replicates, PTI and *I*_PSI_ coefficients for each test are presented in Table 3. In female-choice tests all *I*_PSI_ values were positive and significant except when ST females were tested with Cam males.

**Table 3:** Numbers of observed pair matings and estimates of sexual isolation across experimental designs

Experimental design	Number of replicates	Number of effective matings				*I*_PSI_±1SD	*P*

		Eg Eg	Eg Cam	Cam Eg	Cam Cam		
Male-choice	108	17	23	33	27	−0.126±0.10	0.222
Female-choice	142	36	19	15	43	0.402±0.09	<0.001
Multiple-choice	53	12	8	7	24	0.397±0.14	0.008
		Eg Eg	Eg ST	ST Eg	ST ST		
Male-choice	106	14	14	36	36	0.001±0.11	0.996
Female-choice	127	46	9	35	17	0.222±0.11	0.046
Multiple-choice	53	13	5	13	22	0.355±0.13	0.015
		ST ST	ST Cam	Cam ST	Cam Cam		
Male-choice	112	32	22	18	28	0.205±0.10	0.038
Female-choice	124	6	44	4	46	−0.110±0.17	0.499
Multiple-choice	55	11	14	11	14	0.124±0.15	0.409

Male-choice, Female-choice, and multiple-choice are compared for each cross. In pairings, species of female is given first. *I*_PSI_ coefficients, their standard deviations and their significance of deviation from the null hypothesis (i.e., random mating) were calculated in JMATING by resampling the observed values 10,000 times.

The PTI values were significantly greater than 1 for Eg and Cam intra-strain matings, showing that Eg and Cam females preferred to mate with their own males. ST females preferred Eg (PTI = 1.4, *P* = 0.02) and Cam males (PTI = 1.8, *P* < 0.001). We did not observe any male preference for a specific female, except for Eg males which preferred ST females to their own females, as previously observed in the no-choice experiments. ST males also copulated more with their own females, leading to a positive and significant *I*_PSI_ value when mated with Cam females. In multiple-choice mating trials a high proportion of males (over 66%) copulated with a female belonging to their own strain. We observed premating isolation between Eg strain and Cam (*I*_PSI_ = 0.40, *P* < 0.01) and Eg and ST (*I*_PSI_ = 0.36, *P* < 0.05). We observed no premating isolation between Cam and ST.

## Discussion

*Drosophila simulans* originated in coastal eastern Africa and/or islands in the Indian Ocean. It has undergone demographic expansion, to the West and following along the Nile route presumably after the last ice age, about 10 000 years ago (Lachaise et al. 1988). *Drosophila simulans* is considered a cosmopolitan species although its distribution is not uniform. It is widespread in Africa, and abundant in central and east Africa, but absent from most of West Africa. In West Africa, *D. simulans* is found mostly in Cameroon but not to the west of the Cameroon volcanic line (Lachaise et al. 1988). Molecular polymorphism at four X-linked genes suggests that populations from East and continental Africa show little differentiation, Cameroon population markedly differentiated from the others (Baudry et al. 2006). In fact, the Cameroon population has previously been shown to differ in its HC pattern (Rolan-Alvarez and Caballero 2000; Rouault et al. 2004). Our present study shows partial premating isolation between Eg (7-T) flies and ST and Cam (I and 7-P) flies. This isolation seems to be caused by a clear preference of Eg and Cam females for their own males. Eg and Cam flies leave in different geographic locations and reproductive isolation may have evolved indirectly as a consequence of divergent natural selection on these populations. This study focuses only on premating isolation, which involves mostly pheromones (which also depend on genetic divergence), and not on postmating isolation, which depends on genetic divergence.

The same HCs occur in male and female *D. simulans* but HCs profiles are quantitatively sexually dimorphic (Ferveur 1991; Ferveur and Jallon 1993) and the variations of their profiles are heritable (Sharma et al. 2012a). Here, in accordance with literature, we observe that HC composition differs markedly between males and females. In all three types of strains, females had more 7-T than males, and Cam and ST females synthesized less 7-P than males. We also observed longer chain (C29) HCs in females than in males, especially in Eg females.

Female HC composition is known to modulate male courtship latency. In *D. melanogaster*, 7,11-heptacosadiene is the most potent female pheromone, acting at the nanogram level (Ferveur and Sureau 1996) although mono- and di-unsaturated HCs with 27 ± 2 carbons are also involved in courtship behavior (Antony et al. 1985). In *D. simulans*, courtship experiments have suggested that both 7-T and 7-P could induce courtship (Cobb and Jallon 1990); however, the role of these HCs is still not clear. In our study, ST males were more attracted to their own females, Eg males seemed to prefer Cam and ST females and Cam males had no preference. This shows the complexity of pheromone interactions and male receptivity in *D. simulans*. The 7-T could help males to recognize females, although other compounds like methyl hexacosane and methyl octacosane might also play a role.

Sexual selection is stronger in males than in females; it is known to be costly and non adaptative in *D. simulans* (Sharma et al. 2012b) and other *Drosophila* species (Holland 2002). However, female mate preference has been poorly studied in *D. simulans* especially for males with different pheromonal profiles. In this study, we describe partial reproductive isolation between Eg (7-T) flies and ST and Cam (I and 7-P) flies, due to a preference of Eg and Cam females for their own males. The lack of reproductive isolation between ST and Cam populations might be a consequence of their quite very similar HC profiles.

The effect of natural selection (through temperature and desiccation) on HCs has been studied but is not fully understood. In *D. melanogaster*, selection for resistance to desiccation is accompanied by changes in HC pattern, generally an increase in chain length (Kwan and Rundle 2010; Foley and Telonis-Scott 2011). In *D. simulans*, a preliminary study failed to find a relationship between 7-T/7-P ratio and desiccation resistance (Rouault et al. 2004). However, this study measured desiccation at 32°C, a very high temperature at which flies die rapidly. In the current study, there was no correlation either between HC profiles and resistance to desiccation at 25°C.

The 7-P phenotype in Cam and ST males is intriguing; it seems to offer them only slightly higher resistance to desiccation than Eg males, although Eg males have fewer cuticular HCs overall. Surprisingly, Eg females were the most resistant to desiccation, although their 7-P percentage was ten times lower than females of the other strains. Eg females had twice as much 2 methyloctacosane as other females, which might be one reason for their heightened resistance. In this respect, *D. simulans* seems very different from *D. melanogaster*. First, *D. melanogaster* is twice resistant to desiccation at 25°C than *D. simulans* (Bontonou et al., unpublished data and this study). Kellermann et al. (2009) also found that *D. simulans* was less tolerant to desiccation than *D. melanogaster*. Second, previous studies have linked desiccation resistance to hydrocarbon composition in *D. melanogaster* (Rouault et al. 2004; Foley and Telonis-Scott 2011) but this link does not exist in *D. simulans*. In *D. melanogaster*, other factors can modify this resistance, such as water loss, water content (Gibbs et al. 1997; Bazinet et al. 2010), lipids (Clark and Doane 1983; van Herrewege and David 1997) and carbohydrates (Chippindale et al. 1998; Gefen et al. 2006). Data for *D. simulans* are more limited. Carbohydrates are known to differ between *D. simulans* strains that also differ in their ability to resist desiccation (Gefen and Brendzel 2011. Interestingly, Gefen and Brendzel (2011) report that viability of female *D. simulans* is more than double that of males in desiccant conditions. Female *D. simulans* also carry more HCs, suggesting again that the absolute amount of HCs is an important factor for surviving desiccation. Hoffmann and Parsons (1989) suggest that desiccation resistance is highly heritable in *D. melanogaster*, unlike *D. simulans*. Interestingly, resistance to desiccation in a temperate population of *D. simulans* showed a certain plasticity; it was increased as the temperature increased, and decreased as the temperature decreased (McKenzie and Parsons 1974). In our study, lower temperature led to a decrease in hydrocarbon production, one factor important for preventing water loss through the cuticle. Contrary to *D. melanogaster* males, in *D. simulans* males, higher temperatures affected most of the HCs- especially branched ones- and not just the monoenes with 23 and 25 carbons.

This study demonstrates partial reproductive isolation of *D. simulans* populations and stresses the importance of male pheromones in this process-a phenomenon that has also recently been demonstrated in *D. melanogaster* (Grillet et al. 2012). We are currently searching for the gene(s) involved in male pheromone phenotype.

## Supporting Information

**Table S1.** Geographical strains of Drosophila simulans.

**Table S2.** Analysis of differences between the HC profiles of males from the Eg strain at three temperatures.

**Table S3.** Analysis of differences between the HC profiles of males from the ST strain at three temperatures.

**Table S4.** Analysis of differences between the HC profiles of males from the Cam strain at three temperatures.

**Table S5.** Analysis of differences between the HC profiles of females from the Eg strain at three temperatures.

**Table S6.** Analysis of differences between the HC profiles of females from the ST strain at three temperatures.

**Table S7.** Analysis of differences between the HC profiles of females from the Cam strain at three temperatures.
