# Supplementary material for: Male pheromone polymorphism and reproductive isolation in populations of Drosophila simulans
Source: Ecol Evol. 2012 Sep 8;2(10):2527–36. doi: 10.1002/ece3.342 (PMC3492778; doi:10.1002/ece3.342)
Supplement: Supplementary file 1 [file ece30002-2527-SD1.docx]

Supplementary Table 1. Geographical strains of *D. simulans*. For each strain, the place where the strain was collected, the year of collection, the number N of males, the percentages of 7-T and 7-P and the 7-T/7-P ratio are mentioned.

| Name | Place | Data of  collection | N | 7-T (%) | | 7-P (%) | | 7-T/7-P |
| --- | --- | --- | --- | --- | --- | --- | --- | --- |
|  |  |  |  | mean | SEM | mean | SEM | mean |
| Ri20 | Brasil (Rio) | 1999 | 5 | 47.03 | 0.92 | 4.85 | 0.21 | 9.69 |
| Ri33 | Brasil (Rio) | 1999 | 5 | 49.14 | 0.59 | 8.81 | 0.28 | 5.58 |
| At8 | Greece (Athens) | 2008 | 5 | 54.62 | 1.93 | 5.04 | 0.34 | 10.83 |
| At9 | Greece (Athens) | 2008 | 5 | 56.43 | 0.15 | 3.93 | 0.15 | 14.37 |
| E6 | Israel (Eilat) | 2000 | 5 | 41.54 | 2.93 | 7.08 | 0.31 | 5.87 |
| E9 | Israel (Eilat) | 2000 | 5 | 50.69 | 1.73 | 6.47 | 0.52 | 7.83 |
| Al12 | Egypt (Alexandria) | 2006 | 10 | 52.01 | 1.13 | 4.08 | 0.17 | 12.74 |
| Al15 | Egypt (Alexandria) | 2006 | 5 | 44.33 | 1.71 | 6.25 | 0.50 | 7.09 |
| K11 | Kenya (Nairobi) | 2001 | 5 | 45.98 | 2.62 | 5.16 | 0.31 | 0.90 |
| K23 | Kenya (Nairobi) | 2001 | 5 | 50.38 | 0.68 | 8.02 | 0.15 | 6.28 |
| Ma238 | Madagascar | 2008 | 5 | 50.68 | 0.52 | 5.86 | 0.19 | 8.65 |
| Ma239 | Madagascar | 2008 | 5 | 50.24 | 1.69 | 4.89 | 0.39 | 10.27 |
| Dz6 | France (Mayotte) | 1999 | 5 | 50.41 | 1.47 | 4.60 | 0.55 | 10.95 |
| Dz9 | France (Mayotte) | 1999 | 5 | 50.89 | 2.59 | 4.26 | 0.21 | 11.96 |
| Rf25 | France (Mayotte) | 2009 | 5 | 54.63 | 1.52 | 3.97 | 0.37 | 13.76 |
| Rf26 | France (Mayotte) | 2009 | 5 | 47.77 | 2.05 | 3.75 | 0.17 | 12.75 |
| Ch001 | Mozambique | 2007 | 5 | 46.48 | 2.85 | 11.96 | 3.52 | 3.89 |
| Ki52 | Ouganda | 2003 | 5 | 57.38 | 0.74 | 6.51 | 0.21 | 8.82 |
| Ki53 | Ouganda | 2003 | 5 | 40.81 | 2.84 | 14.70 | 0.90 | 2.78 |
| Ki61 | Ouganda | 2003 | 5 | 51.68 | 1.14 | 7.20 | 0.20 | 7.18 |
| Sa22 | France (Reunion) | 1999 | 5 | 49.43 | 4.91 | 3.73 | 0.19 | 13.24 |
| Sa32 | France (Reunion) | 1999 | 5 | 53.44 | 2.90 | 2.81 | 0.50 | 19.03 |
| Ru9 | France (Reunion) | 2009 | 5 | 51.29 | 1.28 | 5.99 | 0.30 | 8.56 |
| Ru19 | France (Reunion) | 2009 | 5 | 53.97 | 1.50 | 3.88 | 0.23 | 13.91 |
| Sey1 | Seychelles | 2003 | 5 | 42.44 | 2.66 | 5.68 | 0.31 | 7.47 |
| Sey3 | Seychelles | 2003 | 5 | 47.72 | 2.08 | 6.02 | 0.65 | 7.93 |
| T2 | Tanzania | 1996 | 5 | 45.25 | 1.39 | 7.59 | 0.41 | 5.96 |
| T21 | Tanzania | 1996 | 5 | 53.19 | 2.02 | 3.73 | 0.21 | 14.28 |
| N2-12 | Tunisia (Nasrala) | 1998 | 5 | 56.12 | 0.67 | 3.33 | 0.49 | 16.87 |
| N2-14 | Tunisia (Nasrala) | 1998 | 5 | 49.83 | 3.79 | 5.17 | 0.83 | 9.64 |
| Z5-8 | Zimbabwe | 1997 | 5 | 52.86 | 2.09 | 4.95 | 0.16 | 10.69 |
| BS1 | Sao Tome | 2001 | 5 | 17.93 | 0.49 | 32.13 | 0.35 | 0.56 |
| BS3 | Sao Tome | 2001 | 10 | 20.42 | 0.56 | 34.55 | 0.87 | 0.59 |
| 386-7 | Cameroon | 2004 | 5 | 8.95 | 0.47 | 37.48 | 0.78 | 0.24 |
| 386-11 | Cameroon | 2004 | 10 | 14.93 | 0.47 | 39.15 | 0.44 | 0.38 |
| 413-4 | Cameroon | 2005 | 5 | 15.49 | 0.23 | 40.09 | 1.29 | 0.39 |
| 413-5 | Cameroon | 2005 | 5 | 9.61 | 0.44 | 46.35 | 0.83 | 0.21 |
