# Supplementary material for: Male pheromone polymorphism and reproductive isolation in populations of Drosophila simulans
Source: Ecol Evol. 2012 Sep 8;2(10):2527–36. doi: 10.1002/ece3.342 (PMC3492778; doi:10.1002/ece3.342)
Supplement: Supplementary file 2 [file ece30002-2527-SD2.docx]

Supplementary Table 2. Analysis of differences between the HC profiles of males from the Eg strain at three temperatures. HC identities are given in the first column; elemental composition is listed as the carbon chain length followed by the number of double bonds. HCs are expressed in ng/ fly (first line) and in percentages. Statistical analysis was performed using a one-way ANOVA followed by Tukey’s multiple comparison post-hoc test. *P* values indicated in the table are uncorrected for multiple comparisons; values in bold indicate significant HC variations with temperature. The last three columns give the mean ± SEM (n=10) of HCs produced by individual 7-day old males at 21°C or 5-day old males at 25°C and 29°C.

| **CHC** | ***F*** | ***P*** | **21°C** | **25°C** | **29°C** |
| --- | --- | --- | --- | --- | --- |
| HC (ng/fly) | 3.62 | 0.04 | 1201±73 | 1577±78 | 1450±138 |
| 2-Me-C22 | 0.22 | 0.80 | 0.19±0.03 | 0.17±0.04 | 0.24±0.05 |
| (Z)-9-C23:1 | 9.57 | <.001 | 4.17±0.14 | 5.24±0.21 | 4.29±0.206 |
| (Z)-7-C23:1 | 3.83 | 0.03 | 55.43±0.86 | 52.01±1.13 | 55.91±1.22 |
| (Z)-5-C23:1 | 5.67 | **0.009** | 2.65±0.12 | 2.08±0.08 | 2.19±0.11 |
| C23 | 7.66 | **0.002** | 10.71±0.35 | 12.78±0.42 | 11.89±0.34 |
| 2-Me-C24 | 66.97 | **<.0001** | 3.36±0.21 | 0.94±0.19 | 0.60±0.10 |
| (Z)-9-C25:1 | 16.89 | **<.0001** | 1.59±0.12 | 2.62±0.13 | 2.18±0.13 |
| (Z)-7-C25:1 | 3.72 | 0.04 | 3.32±0.28 | 4.08±0.17 | 3.52±0.13 |
| (Z)-5-C25:1 | 0.06 | 0.94 | 0.08±0.03 | 0.05±0.02 | 0.09±0.06 |
| C25 | 6.06 | 0.007 | 2.33±0.23 | 3.09±0.27 | 3.62±0.33 |
| 2-Me-C26 | 66.53 | **<.0001** | 11.98±0.49 | 7.40±0.44 | 4.26±0.47 |
| C27 | 11.35 | **<.001** | 1.06±0.17 | 2.10±0.32 | 3.21±0.29 |
| 2-Me-C28 | 73.59 | **<.0001** | 2.37±0.20 | 6.20±0.26 | 6.58±0.41 |
| C29 | 1.75 | 0.19 | 0.71±0.19 | 0.45±0.10 | 0.70±0.08 |
