# Supplementary material for: Male pheromone polymorphism and reproductive isolation in populations of Drosophila simulans
Source: Ecol Evol. 2012 Sep 8;2(10):2527–36. doi: 10.1002/ece3.342 (PMC3492778; doi:10.1002/ece3.342)
Supplement: Supplementary file 3 [file ece30002-2527-SD3.docx]

Supplementary Table 3. Analysis of differences between the HC profiles of males from the ST strain at three temperatures. HC identities are given in the first column; elemental composition is listed as the carbon chain length followed by the number of double bonds. HCs are expressed in ng/ fly (first line) and in percentages. Statistical analysis was performed using a one-way ANOVA followed by Tukey’s multiple comparison post-hoc test. *P* values indicated in the table are uncorrected for multiple comparisons; values in bold indicate significant HC variations with temperature. The last three columns give the mean ± SEM (n=10) of HCs produced by individual 7-day old males at 21°C or 5-day old males at 25°C and 29°C.

| **CHC** | ***F*** | ***P*** | **21°C** | **25°C** | **29°C** |
| --- | --- | --- | --- | --- | --- |
| HC (ng/fly) | 6.75 | <.01 | 863±32 | 1217±115 | 904±49 |
| 2-Me-C22 | 0,6 | 0,56 | 0.16±0.08 | 0.17±0.05 | 0.11±0.05 |
| (Z)-9-C23:1 | 5,52 | <.01 | 1.04±0.04 | 1.12±0.10 | 0.81±0.07 |
| (Z)-7-C23:1 | 0,85 | 0,45 | 21.64±0.76 | 20.42±0.56 | 21.33±0.72 |
| (Z)-5-C23:1 | 8,01 | <.01 | 1.01±0.06 | 0.88±0.10 | 1.38±0.09 |
| C23 | 8,66 | <.01 | 6.43±0.53 | 7.77±0.21 | 8.71±0.41 |
| 2-Me-C24 | 22,59 | <.0001 | 2.02±0.32 | 0.77±0.09 | 0.58±0.06 |
| (Z)-9-C25:1 | 30,1 | <.0001 | 3.91±0.18 | 5.33±0.18 | 5.47±0.11 |
| (Z)-7-C25:1 | 13,050 | <.001 | 28.95±1.33 | 34.55±0.87 | 35.59±0.61 |
| (Z)-5-C25:1 | 54,420 | <.0001 | 2.36±0.12 | 1.66±0.09 | 0.84±0.09 |
| C25 | 5,550 | <.01 | 3.81±0.51 | 4.14±0.18 | 5.15±0.11 |
| 2-Me-C26 | 89,720 | <.0001 | 14.95±0.63 | 9.55±0.44 | 7.08±0.16 |
| C27 | 18,09 | <.0001 | 1.94±0.45 | 1.51±0.13 | 3.28±0.15 |
| 2-Me-C28 | 0,37 | 0,7 | 3.78±0.51 | 3.88±0.53 | 5.17±0.20 |
| C29 | 61,59 | <.0001 | 0.75±0.28 | 0.66±0.36 | 0.46±0.05 |
