# Supplementary material for: Male pheromone polymorphism and reproductive isolation in populations of Drosophila simulans
Source: Ecol Evol. 2012 Sep 8;2(10):2527–36. doi: 10.1002/ece3.342 (PMC3492778; doi:10.1002/ece3.342)
Supplement: Supplementary file 4 [file ece30002-2527-SD4.docx]

Supplementary Table 4. Analysis of differences between the HC profiles of males from the Cam strain at three temperatures. HC identities are given in the first column; elemental composition is listed as the carbon chain length followed by the number of double bonds. HCs are expressed in ng/ fly (first line) and in percentages. Statistical analysis was performed using a one-way ANOVA followed by Tukey’s multiple comparison post-hoc test. *P* values indicated in the table are uncorrected for multiple comparisons; values in bold indicate significant HC variations with temperature. The last three columns give the mean ± SEM (n=10) of HCs produced by individual 7-day old males at 21°C or 5-day old males at 25°C and 29°C.

| **CHC** | ***F*** | ***P*** | **21°C** | **25°C** | **29°C** |
| --- | --- | --- | --- | --- | --- |
| HC (ng/fly) | 2.82 | 0.08 | 973±55 | 1128±91 | 1270±110 |
| (Z)-9-C23:1 | 1.72 | 0.20 | 1.28±0.10 | 1.05±0.08 | 1.24±0.10 |
| (Z)-7-C23:1 | 15.11 | <.0001 | 20.91±0.78 | 14.93±0.47 | 18.69±1.06 |
| (Z)-5-C23:1 | 18.02 | <.0001 | 1.27±0.10 | 0.76±0.05 | 1.41±0.09 |
| C23 | 17.05 | <.0001 | 7.58±0.37 | 8.26±0.24 | 10.30±0.39 |
| 2-Me-C24 | 7.2 | <.01 | 1.39±0.19 | 0.44±0.07 | 1.15±0.37 |
| (Z)-9-C25:1 | 21.02 | **<.0001** | 4.09±0.25 | 5.88±0.16 | 5.07±0.16 |
| (Z)-7-C25:1 | 14.840 | <.0001 | 34.45±0.80 | 39.15±0.44 | 31.33±1.47 |
| (Z)-5-C25:1 | 26.080 | <.0001 | 1.28±0.07 | 1.09±0.04 | 0.56±0.08 |
| C25 | 7.840 | <.01 | 4.48±0.26 | 5.45±0.16 | 5.55±0.22 |
| 2-Me-C26 | 44.100 | **<.0001** | 12.16±0.28 | 7.02±0.15 | 9.05±0.61 |
| (Z)-9-C27:1 | 2.29 | 0.12 | 0.18±0.03 | 0.31±0.07 | 1.07±0.59 |
| (Z)-7-C27:1 | 27.17 | <.0001 | 1.99±0.21 | 6.87±0.34 | 3.48±0.22 |
| C27 | 14.71 | <.001 | 2.00±0.21 | 3.46±0.26 | 3.80±0.33 |
| 2-Me-C28 | 8.02 | 0.01 | 3.25±0.33 | 4.47±0.29 | 5.22±0.51 |
| C29 | 1.29 | <.01 | 0.80±0.12 | 0.59±0.09 | 0.58±0.10 |
