# Supplementary material for: Male pheromone polymorphism and reproductive isolation in populations of Drosophila simulans
Source: Ecol Evol. 2012 Sep 8;2(10):2527–36. doi: 10.1002/ece3.342 (PMC3492778; doi:10.1002/ece3.342)
Supplement: Supplementary file 5 [file ece30002-2527-SD5.docx]

Supplementary Table 5. Analysis of differences between the HC profiles of females from the Eg strain at three temperatures. HC identities are given in the first column; elemental composition is listed as the carbon chain length followed by the number of double bonds. HCs are expressed in ng/ fly (first line) and in percentages. Statistical analysis was performed using a one-way ANOVA followed by Tukey’s multiple comparison post-hoc test. *P* values indicated in the table are uncorrected for multiple comparisons; values in bold indicate significant HC variations with temperature. The last three columns give the mean ± SEM (n=10) of HCs produced by individual 7-day old males at 21°C or 5-day old females at 25°C and 29°C.

| **HC** | ***F*** | ***P*** | **21°C** | **25°C** | **29°C** |
| --- | --- | --- | --- | --- | --- |
| HC (ng/fly) | 0.65 | 0.53 | 2556±169 | 2141±167 | 2374±126 |
| 2-Me-C22 | 13.48 | <.001 | 0.27±0.04 | 0.06±0.03 | 0.06±0.02 |
| (Z)-9-C23:1 | 0.73 | 0.5 | 1.96±0.09 | 2.10±0.08 | 1.92±0.21 |
| (Z)-7-C23:1 | 10.18 | <.01 | 60.65±1.14 | 54.83±1.1 | 55.04±1.09 |
| (Z)-5-C23:1 | 9.92 | <.01 | 2.35±0.07 | 1.96±0.07 | 2.03±0.04 |
| C23 | 1.16 | 0.34 | 10.05±0.42 | 9.97±0.22 | 8.98±0.57 |
| 2-Me-C24 | 144.46 | **<.0001** | 2.88±0.19 | 0.28±0.05 | 0.55±0.02 |
| (Z)-9-C25:1 | 56.040 | **<.0001** | 0.9±0.04 | 2.33±0.08 | 2.89±0.24 |
| (Z)-7-C25:1 | 36.880 | <.0001 | 1.81±0.09 | 3.87±0.28 | 3.62±0.19 |
| (Z)-5-C25:1 | 0.730 | 0.5 | 0.04±0.02 | 0.03±0.01 | 0.02±0.01 |
| C25 | 22.690 | <.0001 | 2.12±0.15 | 3.84±0.28 | 4.78±0.45 |
| 2-Me-C26 | 61.47 | **<.0001** | 10.8±0.4 | 3.8±0.24 | 5.23±0.43 |
| C27 | 41.11 | <.0001 | 1.22±0.15 | 5.38±0.4 | 5.10±0.6 |
| 2-Me-C28 | 53.36 | <.0001 | 4.47±0.32 | 8.89±0.4 | 7.89±0.17 |
| C29 | 44.2 | **<.0001** | 0.53±0.09 | 2.68±0.29 | 1.80±0.13 |
