# Supplementary material for: Male pheromone polymorphism and reproductive isolation in populations of Drosophila simulans
Source: Ecol Evol. 2012 Sep 8;2(10):2527–36. doi: 10.1002/ece3.342 (PMC3492778; doi:10.1002/ece3.342)
Supplement: Supplementary file 6 [file ece30002-2527-SD6.docx]

Supplementary Table 6. Analysis of differences between the HC profiles of females from the ST strain at three temperatures. HC identities are given in the first column; elemental composition is listed as the carbon chain length followed by the number of double bonds. HCs are expressed in ng/ fly (first line) and in percentages. Statistical analysis was performed using a one-way ANOVA followed by Tukey’s multiple comparison post-hoc test. *P* values indicated in the table are uncorrected for multiple comparisons; values in bold indicate significant HC variations with temperature. The last three columns give the mean ± SEM (n=10) of HCs produced by individual 7-day old males at 21°C or 5-day old females at 25°C and 29°C.

| **CHC** | ***F*** | ***P*** | **21°C** | **25°C** | **29°C** |
| --- | --- | --- | --- | --- | --- |
| HC (ng/fly) | 1.31 | 0.28 | 3178±267 | 2767±210 | 2718±177 |
| 2-Me-C22 | 1.03 | 0.38 | 0.07±0.03 | 0.03±0.02 | 0.03±0.02 |
| (Z)-9-C23:1 | 4.57 | 0.02 | 0.62±0.05 | 0.72±0.1 | 0.47±0.02 |
| (Z)-7-C23:1 | 0.02 | 0.99 | 30.38±0.61 | 30.25±0.63 | 30.28±0.79 |
| (Z)-5-C23:1 | 4.03 | 0.03 | 2.28±0.08 | 2.12±0.08 | 2.02±0.05 |
| C23 | 7.58 | <.01 | 6.88±0.25 | 8.04±0.16 | 7.63±0.25 |
| 2-Me-C24 | 70.58 | **<.0001** | 1.65±0.04 | 0.63±0.12 | 0.39±0.04 |
| (Z)-9-C25:1 | 35.10 | **<.0001** | 2.78±0.13 | 3.56±0.15 | 4.24±0.08 |
| (Z)-7-C25:1 | 8.59 | **<.01** | 34.08±0.33 | 29.81±0.98 | 32.60±0.78 |
| (Z)-5-C25:1 | 55.43 | <.0001 | 1.00±0.07 | 0.41±0.04 | 0.38±0.04 |
| C25 | 20.77 | <.0001 | 3.06±0.17 | 5.07±0.38 | 4.98±0.24 |
| 2-Me-C26 | 79.23 | <.0001 | 10.84±0.25 | 6.36±0.32 | 5.59±0.33 |
| C27 | 93.99 | <.0001 | 0.11±0.06 | 0.04±0.02 | 0.07±0.04 |
| 2-Me-C28 | 18.41 | <.0001 | 3.08±0.22 | 4.59±0.26 | 4.46±0.14 |
| C29 | 21.84 | <.0001 | 0.31±0.09 | 2.05±0.44 | 1.15±0.07 |
