# Supplementary material for: Male pheromone polymorphism and reproductive isolation in populations of Drosophila simulans
Source: Ecol Evol. 2012 Sep 8;2(10):2527–36. doi: 10.1002/ece3.342 (PMC3492778; doi:10.1002/ece3.342)
Supplement: Supplementary file 7 [file ece30002-2527-SD7.docx]

Supplementary Table 7. Analysis of differences between the HC profiles of females from the Cam strain at three temperatures. HC identities are given in the first column; elemental composition is listed as the carbon chain length followed by the number of double bonds. HCs are expressed in ng/ fly (first line) and in percentages. Statistical analysis was performed using a one-way ANOVA followed by Tukey’s multiple comparison post-hoc test. *P* values indicated in the table are uncorrected for multiple comparisons; values in bold indicate significant HC variations with temperature. The last three columns give the mean ± SEM (n=10) of HCs produced by individual 7-day old males at 21°C or 5-day old females at 25°C and 29°C.

| **HC** | ***F*** | ***P*** | **21°C** | **25°C** | **29°C** |
| --- | --- | --- | --- | --- | --- |
| HC (ng/fly) | 13.83 | **<.0001** | 2098±104 | 3044±187 | 2021±157 |
| 2-Me-C22 | 1.51 | 0.24 | 0.09±0.04 | 0.03±0.02 | 0.04±0.02 |
| (Z)-9-C23:1 | 7.96 | <.01 | 1.10±0.06 | 0.76±0.11 | 0.68±0.07 |
| (Z)-7-C23:1 | 4.08 | 0.03 | 24.71±1.01 | 26.67±1.42 | 21.28±1.56 |
| (Z)-5-C23:1 | 15.03 | <.0001 | 1.34±0.09 | 1.84±0.16 | 1.01±0.08 |
| C23 | 2.34 | 0.12 | 7.69±0.16 | 7.97±0.16 | 8.74±0.53 |
| 2-Me-C24 | 6.67 | <.01 | 0.91±0.07 | 0.45±0.07 | 0.73±0.12 |
| (Z)-9-C25:1 | 5.84 | <.01 | 3.54±0.24 | 3.87±0.21 | 4.51±0.17 |
| (Z)-7-C25:1 | 0.500 | 0.620 | 34.09±0.87 | 32.6±1.09 | 33.32±1.22 |
| (Z)-5-C25:1 | 62.610 | **<.0001** | 1.03±0.07 | 0.59±0.04 | 0.2±0.06 |
| C25 | 9.750 | <.001 | 4.30±0.20 | 5.28±0.54 | 6.61±0.37 |
| 2-Me-C26 | 11.660 | <.001 | 9.12±0.31 | 6.24±0.17 | 7.36±0.64 |
| (Z)-9-C27:1 | 0.38 | 0.69 | 0.16±0.05 | 0.09±0.03 | 0.12±0.05 |
| (Z)-7-C27:1 | 2.96 | 0.07 | 3.61±0.29 | 2.56±0.34 | 2.5±0.60 |
| C27 | 8.95 | <.01 | 2.58±0.22 | 4.49±0.74 | 5.29±0.54 |
| 2-Me-C28 | 3.94 | 0.04 | 4.75±0.30 | 4.36±0.24 | 5.66±0.44 |
| C29 | 4.07 | 0.03 | 0.86±0.15 | 2.2±0.70 | 1.82±0.22 |
